# The role of intrapersonal and interpersonal factors in waterpipe cessation: a case-control study

**DOI:** 10.1186/s12889-023-16597-5

**Published:** 2023-09-04

**Authors:** Sara Dadipoor, Mojtaba Hemayatkhah, Hadi Eshaghi Sani Kakhaki, Shokrollah Mohseni, Esmaeil Fattahi, Nahid Shahabi, Omar El-Shahawy

**Affiliations:** 1 Tobacco and Health Research Centre, Hormozgan University of Medical Sciences, Bandar Abbas, Bandar Abbas, Iran; 2 Department of Sociology, Payame Noor University, Tehran, Iran; 3 Determinants in Health Promotion Research Centre, Hormozgan Health Institute, Hormozgan University of Medical Sciences, Bandar Abbas, Iran; 4 Department of Health Education and Promotion, School of Health, Guilan University of Medical Sciences, Rasht, Iran; 5 Department of Population Health, New York University Grossman School of Medicine, New York, NY USA

**Keywords:** Cessation, Iran, Self-efficacy, Water-pipe, Women, Subjective norm

## Abstract

**Background:**

The prevalence of waterpipe smoking among women in southern Iran is significantly higher than women in other regions of Iran. We aimed to explore the effect of several demographic factors, knowledge, attitude, self-efficacy and social norms on a successful cessation of waterpipe smoking in the marginalized women of Bandar Abbas city, in the south of Iran.

**Methods:**

This case-control study was conducted in 2022 among 731 women (246 subjects who successfully quit waterpipe smoking in the case group and 485 who smoked waterpipe in the control group). A cluster sampling method was used to collect the required data through face-to-face interviews and a researcher-made questionnaire. The questionnaire consisted of demographic information, behavioral information about waterpipe smoking and knowledge, attitude, self-efficacy and social norms. The data were analyzed in STATA 14 using univariate and multivariate regression analyses.

**Results:**

The mean and standard deviation of age was 39.24 ± 11.93 and 37.18 ± 13.57 in the control and case groups, respectively. With an increase of one score in social norm (OR: 1.046), the odds of cessation were increased for 4%. With an increase of one score in self-efficacy (OR: 1.152), the odds of cessation were increased for 15%. With an increase of one score in knowledge (OR: 1.064), the odds of cessation were increased for 6%. With an increase of one score in attitude (OR: 1.215) the odds of cessation were increased for 21%.

**Conclusion:**

The present findings revealed personal and interpersonal influential factors in successful waterpipe cessation. Women’s knowledge can be increased and their attitude can be changed. Important people in women’s lives can be influenced to, consequently, affect women positively and improve their self-esteem.

## Background

Tobacco smoking is the leading preventable cause of morbidity and mortality globally [1]. Tobacco (smoking, second-hand smoking, and chewing) accounted for around nine million deaths in 2019 [2]. Most (80%) of the world’s smokers dwell in developing countries and in particular in recent decades females are becoming more exposed to tobacco and alcohol nowadays than ever before [3]. Waterpipe smoking, also known as shisha, narghile, hubble-bubble, and hookah, is a form of tobacco smoking that entails the inhaling of heated tobacco smoke after it passes through water [4], and is now increasing in popularity across the globe [5]. It is second most common type of tobacco smoking in many countries, but particularly in the Eastern Mediterranean Region (EMR) [6].

The prevalence of waterpipe smoking among males and females in 2019 was estimated to be, respectively, 32.7% and 46.2% in Lebanon, 13.4% and 7.8% in Jordan, and 18.0% and 7.9% in Palestine [7]. Increasingly, the use of waterpipe, especially among women, has become a normative social behavior [8]. In Iran, 82% of women who smoke tobacco use waterpipes [9]. The prevalence of waterpipe smoking among Iranian women is reported to be between 3.8% and 6.3% [10, 11]. There is regional variation in prevalence among women smokers where those in the south of Iran are 9–10 times as high as women in other provinces [12]. For example, the prevalence of waterpipe smoking in Bandar Abbas city is reported to be 15.1%, and among women it is reported to be higher than men [13, 14], according to the results of the most recent community health assessment (CHA) survey. This high prevalence of waterpipe smoking among women reflects a major public health concern [14].

Waterpipe smoking among women can cause respiratory diseases, lung cancer, mouth cancer [15], infant mortality, increased risk of premature menopause, decreased bone density, infertility, ectopic pregnancy, and genital warts [16–18]. Given this morbidity risk compounded by a high prevalence of waterpipe smoking among women in southern Iran, it is imperative to put every effort to support waterpipe cessation. Several studies demonstrated the key role of knowledge and attitude pertaining to tobacco smoking, especially waterpipe [19, 20]. Thus, a sound knowledge and understanding of the harmful effects of waterpipe that can alter prevailing favorable attitudes can help decrease waterpipe consumption [21]. Similarly, subjective norms (SN, i.e., social influences on behavior) can also impact women’s waterpipe smoking and cessation behavior [22, 23]. Many researchers have pinpointed self-efficacy as a key factor in establishing successful smoking cessation programs [24, 25]. According to Bandura’s theory, self-efficacy is one’s belief in beginning the waterpipe cessation behavior and how long they manage to maintain this behavior in the face of obstacles or adversities [26]. It is suggested that improving each of the above-mentioned factors can enhance the effectiveness of a waterpipe cessation intervention.

There is a paucity of research pertaining to waterpipe cessation, since most published studies focused on intention to quit waterpipe smoking rather than assessing waterpipe quit behaviors [27, 28]. To address this gap, we evaluated the personal factors likely associated with waterpipe smoking cessation in marginal women living in Bandar Abbas, in the south of Iran. We hypothesize that knowledge, attitude, self-efficacy and social norms will be significantly associated with successful waterpipe cessation.

## Methods

### Design and participants

This case-control study was conducted between June and October 2022 among women living on the outskirts of Bandar Abbas city in the south of Iran.

Bandar Abbas, a city with a total population of 530 thousand and about 25 thousand (local residents and workers) in its west suburbs, lies on the north coast of Persian Gulf. This city is a leading economic center of Iran as it hosts important seaports and several major nationwide industries. In recent years, Bandar Abbas has experienced an accelerated urban growth because of its proximity to main commercial seaports and its adjacent multiple industrial zones [29]. The research population consisted of women over 15 years of age visiting healthcare centers on the outskirts of Bandar Abbas city, where there is high prevalence of waterpipe smoking among women, even when compared to men [12, 13]. We compared women living in the outskirts of the city, who reported successfully quitting waterpipe smoking for more than 6 months to women reporting current waterpipe smoking only defined as smoking waterpipe for at least 4 times a week for the past 6 months, while not smoking any other tobacco product.

All participants signed an informed consent to participate in the study, this study was approved by the ethics committee of Hormozgan University of Medical Sciences (# IR.HUMS.REC.1401.415).

### Sample

Inclusion criteria was quitting waterpipe for at least 6 months in the case group, smoking waterpipes at least 4 times a week for 6 months in the control group, living in the outskirts of the city and informed and voluntary consent to participate in the study and ability to read and write.

Exclusion criterion in this study was consumption of other tobacco products and incomplete questionnaires for exclusion in analyses.

The attitude score in the cessation group as the case was 17.4 ± 60 and in the control group was 20.8 ± 55. As the findings of the pilot study showed, using the following formula, the sample size was estimated at 230 for the case group. Considering the possibility of incomplete answers, the sample size of the case group and the control group was, respectively, increased to 250 and 500 (two controls per each case).

Z _1-α/2_: 1.96.

Z_1-β_: 0.84.

$$n=\frac{\left(Z_{1-\frac a2}^2+\;Z_{1-\beta}\right)^2\;\left(S_1^2+S_0^2\right)}{\left(\mu_1\;-\;\mu_0\right)^2}$$

**Fig. 1:**
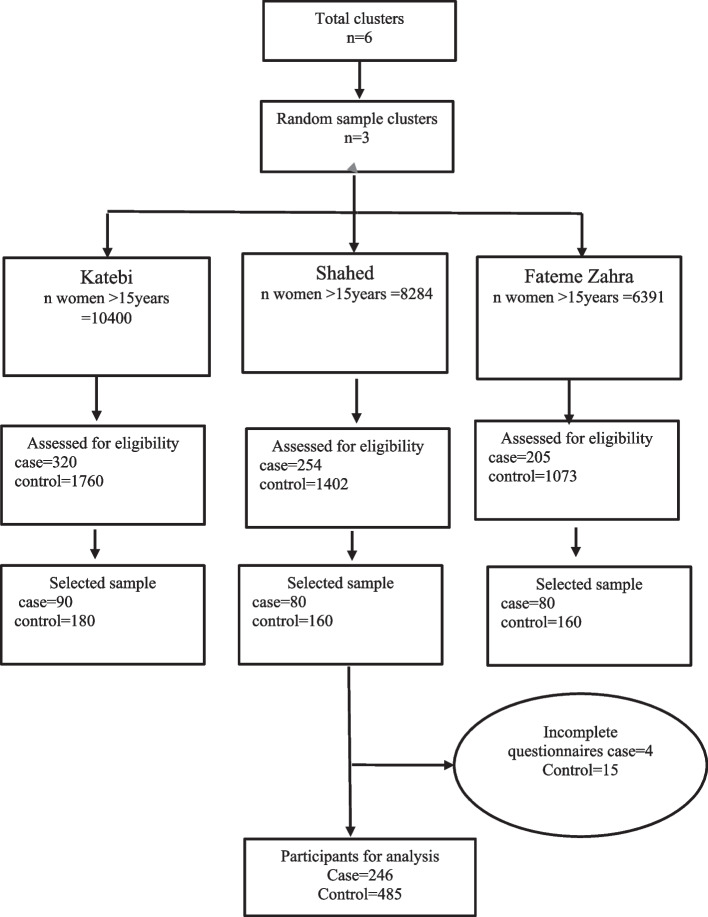
The sampling flowchart

For sample selection, a two-stage cluster sampling method was used. The study included comprehensive healthcare centers on the outskirts of Bandar Abbas city. In the first stage, 3 clusters (Shahed, Fateme Zahra, Katebi) were randomly selected from the 6 comprehensive health healthcare centers on the outskirts of Bandar Abbas city. Then in the second stage, a report was extracted from the national *Seeb* system to select women who were eligible to participate in the study. The output of the *Seeb* was an Excel file. The final output list was divided into two separate Excel files so that the sampling for case and control groups could be done separately. Finally, 80 women were selected from each center for the case group and 160 for the control group. From Katebi health center, 90 women were sampled for the case group and 180 for the control (Fig. 1).

### Measures

The questionnaire was developed based on a review of literature [30, 31]. We included demographic information such as age, marital status, education, occupation. We also included waterpipe smoking behavior, the duration of smoking waterpipes in years, the person supporting the act of cessation (friends, family and the person himself), and the type of waterpipe tobacco consumed (local/fruity/both). The outcome variables were knowledge, attitude, self-efficacy and social norms. Knowledge assessment used a rating of 10 questions with responses of YES/NO/DON’T KNOW, which were scored 1, 0 and 0, respectively. An example of knowledge questions is “The risk of lung cancer is higher in waterpipe smokers than non- waterpipe smokers.“ Items 3, 4 and 5 were reversely scored so that a NO as a correct answer scored 1 and answering YES or DON’T KNOW received 0. The knowledge score ranged between 10 and 20. A sample item was “The waterpipe is less harmful than cigarettes and other tobacco products”.

The attitude was assessed via a rating scale of 15 questions with a score ranging from 15–75. A sample question was “Quitting waterpipe requires a lot of effort and energy”. The social norm was rated along 10 “normative beliefs” questions, such as “The family and friends’ opinion about replacing waterpipe with some healthy amusement matters to me”. There were also 10 “motivation to follow” questions, such as “My friends expect me not to hang out with waterpipe smokers”. There were 20 questions with 20–100 points overall for this variable. Self-efficacy was also rated on 9 items. There were 9–45 points. Attitude and motivation to comply, and self-efficacy questions were rated on a 5-point Likert scale: strongly agree, agree, don’t know, disagree, and strongly disagree. They were scored 1–5, respectively Normative beliefs were also rated on a 5-point Likert scale: absolutely important, important, undecided, unimportant and completely unimportant.

### Data quality assurance

To assess the content validity of the measurement instrument, the questionnaire was provided to 5 experts in health education and their opinions were used to modify the questionnaire. To substantiate the reliability of the instrument, the test-retest method was used. The questionnaire was submitted to 30 subjects (15 cases and 15 controls) from people who had the same conditions as those under study during a two-week interval and on two occasions. It was given in a center that was not among the main sampling centers. After that, each question in the first test was compared with the second test. If the correlation coefficient between the first and second tests in each part was higher than 0.7, the questionnaire was reliable. After that, to estimate the agreement between the test and retest, the ICC was calculated. To calculate the agreement between the mean test scores and the mean retest scores, the estimated ICC value was 0.83 for the case group and 0.86 for the control group, and the questionnaire was reliable.

### Data collection

Having obtained an ethical approval of the research and gained the necessary permission, the researchers visited the health centers. A fully informed consent was provided by the participants. The objectives of study and the procedure were explained to the participants. The control and case groups completed the written consent form. The questionnaires were completed by women as self-reports. Each questionnaire took about 15 min to complete. The completion of the questionnaires was done in person. The women completed the questionnaires at home in their convenience and returned them to the researcher.

### Data management

Quantitative variables included age, knowledge, social norms, attitude, and self-efficacy, which were measured and reported using descriptive statistics (standard deviation, maximum, minimum, mean, range). Qualitative variables (marital status, education, job, history of smoking, supporter, and tobacco type) were measured and reported in frequency and percentage. Bivariate logistic regression and multivariate logistic regression was used to test smoking cessation. The data were statistically analyzed in STATA 14 (*P* < 0.05).

## Results

### Descriptive findings

In this study, 246 subjects were assigned to the case group and 485 to the control. The mean and standard deviation of age in the former was 37.18 ± 13.57 and in the latter 39.24 ± 11.93. Out of 246 subjects in the case group, 68.3% were married and 63.0% were employed. Also, 48.4% of the case group relied on themselves to quit smoking and had no other support, and 69.1% used local tobacco. Out of 485 subjects in the control group, 69.3% were married, 41.6% had primary education, and 49.1% had 1–15 years of waterpipe smoking experience. 60.4% of subjects in the control group were local waterpipe smokers. The details of other demographic features are reported in Table 1.

**Table 1:** Descriptive statistics for the research groups

Variables	Categories	Case N 246(%)	Control N 485(%)	*p*-value
**Age**	Mean ± SD	37.18 ± 13.57	39.24 ± 11.93	0.036
**Marital**	Single/divorced Widow	78(31.7)	149(30.7)	0.786
	Married	168(68.3)	336(69.3)	
**Education**	Primary	85(34.6)	202(41.6)	< 0.001
	Secondary School	58(23.6)	133(27.4)	
	Diploma	65(26.4)	123(25.4)	
	University	38(15.4)	27(5.6)	
**Job**	Housewife	85(34.6)	263(54.2)	< 0.001
	Employed	161(65.4)	222(45.8)	
**History of smoking**	1–15 years	155(63.0)	238(49.1)	< 0.001
	16–30 years	59(24.0)	102(21.0)	
	> 30 years	32(13.0)	145(29.9)	
**Support**	Friends	49(19.9)	118(24.3)	0.323
	herself	119(48.4)	211(43.5)	
	Family	78(31.7)	156(32.2)	
**Tobacco type**	Fruity	40(16.3)	121(24.9)	0.023
	Local	170(69.1)	293(60.4)	
	Local-fruity (both)	36(14.6)	71(14.6)	

**Fig. 2:**
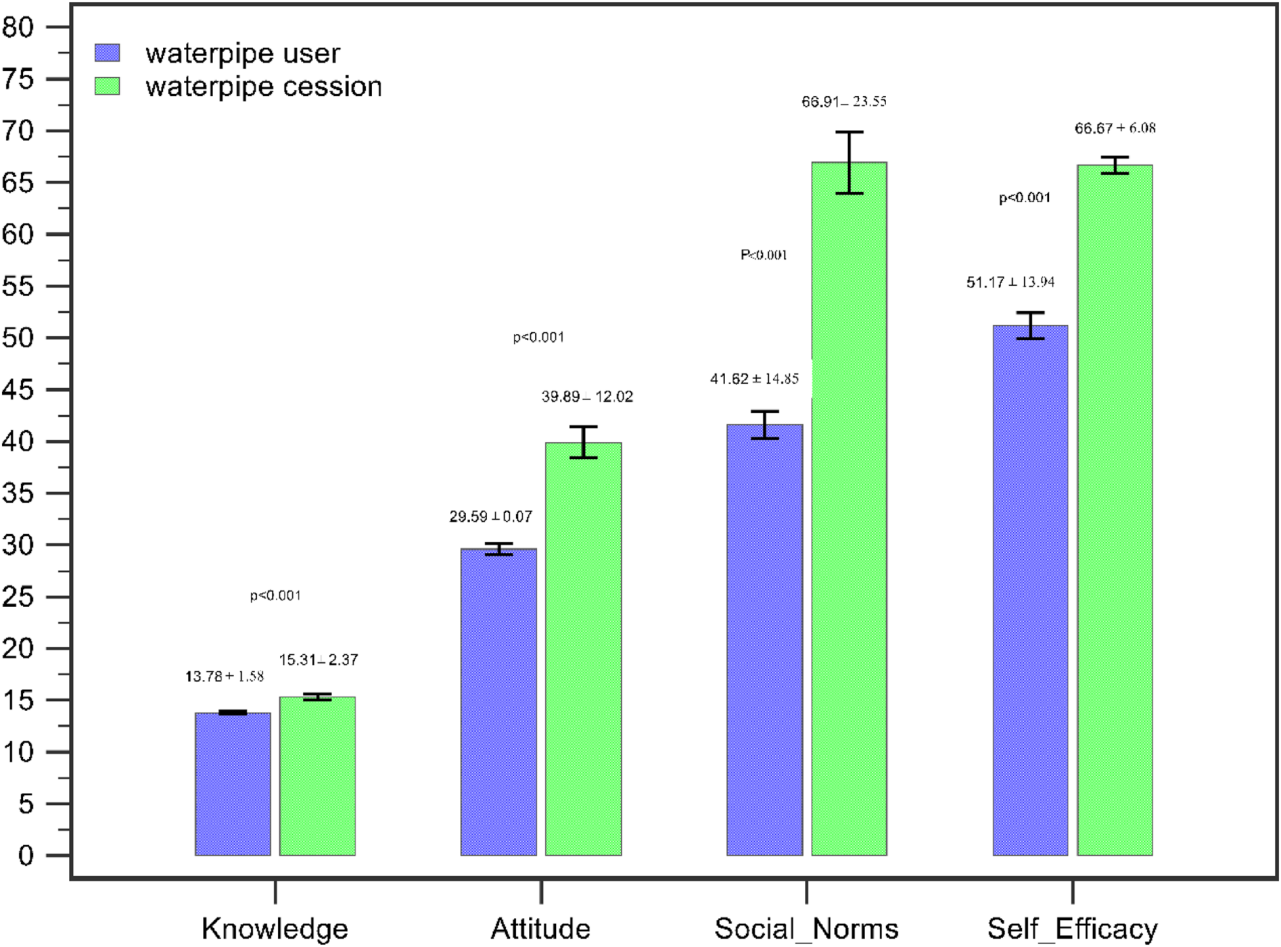
Knowledge, attitude, social norm and self-efficacy scores of case and control groups

Figure 2 shows the mean scores of the group that successfully quit waterpipe smoking (case group) and the group that did not manage to quit (control group) in knowledge, attitude, social norms, and self-efficacy (Fig. 2). As this figure shows, the group in question obtained higher scores of social norms, self-efficacy, attitude and knowledge, and the between-group difference was statistically significant (p < 0.001).

In univariate regression analysis, the odds of quitting waterpipe were 77% lower in women with a > 30-year history of waterpipe smoking than those with 1–15 years of smoking experience. The use of local tobacco increased the odds of cessation by 1.7 times that of the fruity type of tobacco. With an increase of one point in the attitude, the odds of cessation increased for 47%. In the multivariate regression analysis, women with a > 30-year history of smoking waterpipes had 30% lower odds of cessation than those with 1–15 years of smoking. The support of family and friends reduced the odds of cessation by 50% and 54%, respectively, compared to the mere reliance on oneself, with an increase of one score in the attitude, the odds of cessation increased by 21%. The other information is indicated in Table 2.

**Table 2:** Univariate and multivariate logistic regression analyses for waterpipe cessation

		Univariate		Adjusted	
Variable	**Categories**	**OR (95% CI)**	***p* value**	**OR (95% CI)**	***p* value**
**Age**		0.987(0.974-0.999)	0.037	1.057(1.024–1.092)	0.001
**Marital status**	Single/divorced Widow				
	Married	0.955(0.686-1.329)	0.786	0.289(0.153-0.545)	< 0.001
**Education**	Primary				
	Secondary	1.036(0.695-1.545)	0.861	2.379(1.047–5.407)	0.039
	Diploma	1.256(0.848-1.861)	0.256	0.493(0.218-1.112)	0.088
	College	3.345(1.921–5.824)	< 0.001	4.361(1.581–12.027)	0.004
**Job**	Housewife				
	Employed	2.244(1.633–3.083)	< 0.001	5.710(3.091–10.548)	< 0.001
**history of smoking**	1–15				
	16–30	0.888(0.608-1.298)	0.540	0.210(0.090-0.491)	< 0.001
	> 30	0.339(0.220-0.522)	< 0.001	0.072(0.027-0.192)	< 0.001
**Support**	herself				
	Family	0.887(0.623-1.261)	0.503	0.509(0.279-0.928)	0.028
	Friends	0.736(0.493 − 1.100)	0.135	0.454(0.228-0.904)	0.025
**Tobacco type**	Fruity				
	local	1.755(1.171–2.630)	0.006	3.042(1.295–7.349)	0.013
	Local-fruity	1.534(0.896-2.625)	0.119	4.624(1.712–12.488)	0.003
**Social norm**		1.065(1.054–1.075)	< 0.001	1.046(1.028–1.064)	< 0.001
**Self-efficacy**		1.131(1.107–1.156)	< 0.001	1.152(1.116–1.189)	< 0.001
**Knowledge**		1.144(1.117–1.172)	< 0.001	1.064(1.022–1.108)	0.002
**Attitude**		1.474(1.352–1.606)	< 0.001	1.215(1.060–1.393)	0.005

**Fig. 3:**
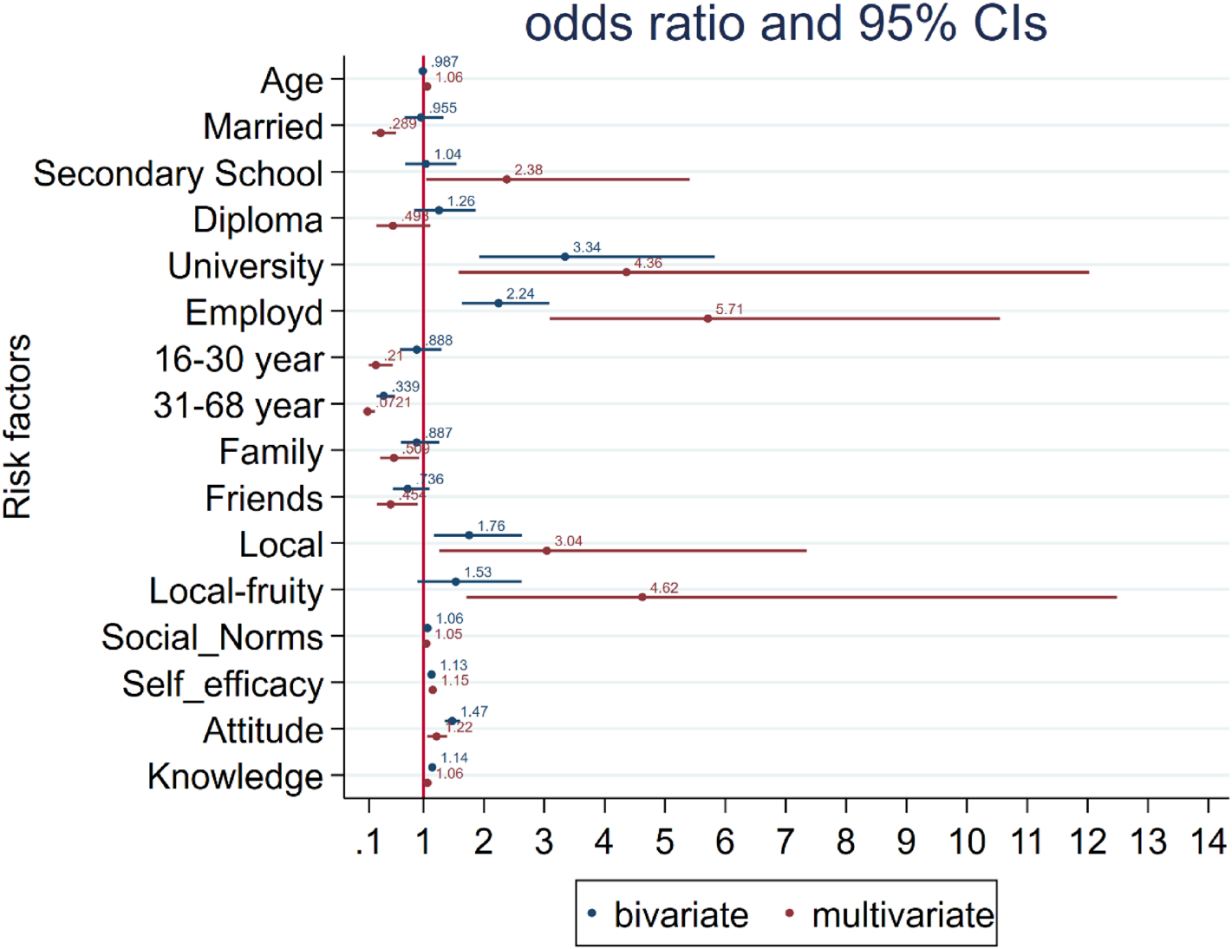
Odds ratios in coefficient plot for bivariate and multivariate regression analysis

The coefficient plot shows the odds ratios of the target variables in univariate and multivariate logistic regression analysis (Fig. 3).

## Discussion

The present research aimed to explore the role of demographic factors, knowledge and attitude, social norms and self-esteem in the waterpipe cessation behavior of women living on the outskirts of Bandar Abbas. As the multivariate regression analysis results showed, attitude, self-efficacy, knowledge and social norms were significantly and positively correlated with waterpipe smoking. Also, there was a statistically significant relationship between the demographic variables of marital status, education, occupation, history of waterpipe smoking, those supporting the smoker, and the type of tobacco.

As the present findings showed, positive attitude as the leading factor increases the chances of quitting waterpipe smoking in women. As the women of the case group who successfully quit waterpipe had a positive view of cessation, they probably perceived the benefits of cessation. In a review article, positive attitude was a main reason for waterpipe smoking in women. In line with the present findings, in other studies, attitude showed to be significantly correlated with smoking cessation [30, 32, 33].Contrary to this research, in a cross-sectional study in Taiwan, attitude was not significantly related to smoking cessation [34]. The difference in demographic characteristics and the tobacco type may be one possible reason for this discrepancy. Therefore, efforts to change the attitude of society members, especially women, towards waterpipe smoking can help successfully quit waterpipe smoking.

In the present research, after attitude, self-efficacy had a significant and strong relationship with waterpipe cessation in women. The case group had more self-efficacy than the control. Self-efficacy, as women’s belief in their capability of successfully quitting smoking, can be a facilitator of successful cessation behavior. Moreover, in this study, those who relied on themselves to quit waterpipes had higher chances of quitting waterpipe than those who relied on family and friend support. It indicates that the effect of self-efficacy is stronger on the cessation behavior. Similarly, other studies found self-efficacy as an important mediator/predictor of smoking cessation [24, 25]. In agreement with this finding, other studies also showed that belief in one’s capabilities is the most important factor in reducing the rate of smoking or the desire to quit smoking [35, 36]. Considering the important role of self-efficacy in successfully quitting waterpipe and the fact that self-efficacy can be improved with appropriate educational interventions [37], it is essential to implement educational interventions for disadvantaged groups of society with a lower self-efficacy to deal with the temptations of smoking waterpipes. In order to increase the self-efficacy of persuading and reinforcing behavior, improving the physical and mental state, it is suggested to model the step-by-step demonstration project and recorded videos on how to resist peer pressure.

As the present study showed, there was a statistically significant relationship between knowledge and waterpipe cessation. Participants in the case group had more knowledge than the control group. Arguably, a great knowledge of the potential threats of waterpipe smoking plays an important role in cessation. In this regard, Sakka drew attention to the role of knowledge in smoking, and contended that low education on nicotine replacement therapy products can be a barrier to cessation [38]. In agreement with this finding, another study in Greece showed a lacking knowledge of the harmful effects of smoking mainly among those less motivated to quit smoking [39]. Therefore, raising women’s awareness of the potential harms of waterpipe smoking and the benefits of cessation should be a priority for health policy-makers.

In the present study, social norm was the last determinant of the cessation behavior in women. Social norms ranked last among the determinants of the waterpipe cessation behavior. The reason might be that the people surrounding women smokers were mostly waterpipe smokers who failed to set any helpful example for cessation. The significant relationship between social norms and waterpipe cessation behavior shows that the significant others in someone’s life and the motivation to follow them facilitate waterpipe cessation. In line with this finding, in another study, the advice of important people significantly affected the intention to quit smoking [40, 41]. In another study, peer educators trained in a smoking cessation program showed to have an important effect on supporting and improving the processes of cognitive and behavioral change in smoking cessation [42]. In some research, attention was drawn to the importance of learning to resist the peer pressure to smoke waterpipe [30]. Therefore, effective variables such as perceived social pressure regarding behavior change should not be neglected. In programs aiming to quit smoking, especially waterpipe, the reaction of important others and influential characters in society need to be considered.

As the present study showed, the odds of quitting waterpipe in working women were 5 times as high as housewives. A qualitative study shows that women’s employment is a leading factor to encourage them to quit and reduce the rate of waterpipe smoking [22]. Unemployment reduces the odds of quitting smoking. Conversely, having fun in a professional job can increase the odds of quitting smoking [43]. Unemployed women (housewives) spend long hours of the day on their own showing no active presence in society, which can probably tempt them to smoke waterpipes. Policymakers’ efforts to initiate home businesses for women, and flourish women’s talents and capacities and their employment can be an effective solution.

As the present findings showed, the odds of quitting waterpipe in individuals with a long history of waterpipe smoking were lower than those with a short history of waterpipe smoking. Probably, the long history of waterpipe consumption formed a habit and physical and mental dependence in women, and breaking this habit was more difficult for those with a longer history of waterpipe smoking. The effective role of a shorter smoking history in successful tobacco cessation was also raised in a review article [44]. Therefore, it is recommended not to neglect the influencing variable of waterpipe habit and physical and psychological dependence on successful waterpipe cessation. There are probably many variables involved in the formation of waterpipe smoking habit, and it is necessary to consider these factors to take an effective measure in improving women’s health by breaking the habit.

As the present study showed, the odds of successful cessation in those with a university degree were 4 times as high as those without. Arguably, the highly educated probably have more access to more information sources, so they gain more knowledge about the disadvantages of waterpipe smoking. Similarly, another study reported that the education level was significantly correlated with the knowledge of disadvantages of smoking. Thus, women with a higher level of literacy were more concerned about the adverse effects of smoking on their health [45]. It is, thus, suggested to plan interventions according to the target population’s demographic features. Those with a lower level of education require more information guidance. and informational assistance is suggested for people with a lower literacy level. Also, improving the health literacy of underprivileged populations should be a priority for health policy makers.

### Implications

The present findings can facilitate the design of systematic and effective interventions to quit waterpipe smoking. It will act as the basis for comparison with the results of future research on this domain. The present findings can help policymakers and health workers find the best effective solutions to quit smoking.

### Strengths and limitations of study

There were certain limitations in this study. One limitation of this study was that the sample selection was unmatched. As the survey were completed in the form of self-report, there was a possibility that the participants would give socially favorable answers, but the researcher tried to minimize the effect of this bias by ensuring the confidentiality of the information. In this research, the male population was not included, so the generalization of the results can only be done to women. Despite the limitations, the current research had strengths too. First of all, it was pioneering in measuring the actual behavior of smoking waterpipes among women in the outskirts of city by including a control group. It can provide us with more accurate and realistic findings; therefore, in a short time and with minimal efforts and costs, we were able to explore the main determinants of waterpipe cessation.

## Conclusion

The present study explored the demographic, social and psychological factors influencing the successfully waterpipe cessation. The primary finding was that besides increasing women’s knowledge and changing their attitude, through influencing important others in their lives and improving the self-esteem, an effective step can be taken to help them successfully quit smoking. In this regard, improving the ability to say no and media planning to create a negative attitude towards waterpipe smoking in society ad making waterpipe smoking sound unpleasant can help reduce the rate of waterpipe smoking even more. There are hopes that the present findings can help health policy makers find effective solutions to smoking, especially waterpipe smoking, and take effective measures to improve women’s health as far as possible.

## Data Availability

The datasets generated during and/or analyzed during the current study are available from the corresponding author on reasonable request.
